# Association between preoperative diaphragmatic dome height for overall survival in patients with lung cancer and obstructive ventilatory disorder

**DOI:** 10.1007/s10147-026-03022-1

**Published:** 2026-04-21

**Authors:** Masaya Noguchi, Yuji Higashimoto, Toshiki Takemoto, Masashi Shiraishi, Hiroki Mizusawa, Kengo Kanki, Ryota Matsuzawa, Akira Tamaki, Yasuhiro Tsutani

**Affiliations:** 1 Department of Rehabilitation, Kindai University Hospital, Sakai City, Osaka 590-0197, Japan; 2 Graduate School of Rehabilitation Science, Hyogo Medical University, Kobe City, Hyogo 650-8530, Japan; 3 Department of Rehabilitation Medicine, Faculty of Medicine, Kindai University, Sakai City, Osaka 590-0197, Japan; 4 Division of Thoracic Surgery, Department of Surgery, Faculty of Medicine, Kindai University, Sakai City, Osaka 590-0197, Japan

**Keywords:** Lung cancer, Obstructive ventilatory disorder, Chest radiograph, Diaphragmatic dome height, Postoperative overall survival, Disease-specific survival

## Abstract

**Background:**

To investigate the relationship between preoperative diaphragmatic dome height (DDH), measured on chest radiographs, and postoperative overall survival (OS) and disease-specific survival (DSS) due to respiratory-related deaths in patients with lung cancer and obstructive ventilatory disorder (OVD).

**Methods:**

This single-center retrospective study included 302 patients with lung cancer and OVD who underwent lobectomy between 2017 and 2024. DDH was measured on chest radiographs 1 month preoperatively, and the patients were divided into the low DDH (lower than the first quartile, 18.8 mm) and high DDH (≥ 18.8 mm) groups. The associations of DDH with OS and DSS were evaluated using Cox proportional hazards models and Fine–Gray competing risk analysis, respectively. Kaplan–Meier curves and log-rank tests were used for survival comparisons.

**Results:**

Overall, 65 patients (21%) died postoperatively. Cox proportional hazards and Fine–Gray analyses indicated that DDH (hazard ratio: 2.10, 95% confidence interval [CI]: 1.22–3.61, *p* < 0.01; subdistribution hazard ratio: 2.50, 95% CI: 1.14–5.45, *p* < 0.05) was an independent prognostic factor for 3-year OS and DSS. Furthermore, survival curve analysis demonstrated that the low DDH group had significantly lower 3-year OS (70% vs 85%, *p* < 0.01) and 3-year DSS (80% vs 92%, *p* < 0.01) compared with the high DDH group.

**Conclusions:**

DDH is an independent prognostic factor for OS and DSS in patients with lung cancer and OVD, suggesting that it can serve as a novel physiological marker for long-term prognosis.

## Introduction

Lung cancer is a leading cause of cancer-related death and among the most common malignant tumors worldwide, with the highest incidence and mortality rates [1]. Surgical resection is the most effective curative treatment for lung cancer [2], and early-stage diagnosis may yield a favorable prognosis [3]. Recently, advances in diagnostic techniques and therapeutic strategies have contributed to improved postoperative survival rates in patients with lung cancer [4, 5]. However, regardless of the preoperative disease stage, various factors including postoperative pulmonary complications (PPCs), poor preoperative surgical tolerance, and insufficient postoperative self-management influence postoperative prognosis [6], resulting in substantial inter-individual variability in long-term outcomes. Therefore, accurately assessing and predicting factors that affect PPC development and long-term survival from the preoperative stage is crucial for optimizing treatment strategies and risk stratification.

Pathological tumor characteristics and various host factors such as age, sex, comorbidities, pulmonary function, nutritional status, and exercise tolerance are the determinants of postoperative survival [7–9]. Patients with lung cancer and obstructive ventilatory disorder (OVD) are prone to pulmonary hyperinflation and reduced respiratory reserve capacity due to emphysematous changes in the lung parenchyma and airway narrowing. These changes reduce surgical tolerance and significantly increase the risk of PPCs and poor long-term survival [10]. Consequently, identifying objective preoperative indicators that reflect the morphological and functional alterations associated with OVD is essential for predicting postoperative prognosis. In particular, diaphragmatic dysfunction is closely related to both smoking and OVD [11]. Recently, the usefulness of preoperative diaphragmatic function assessment in thoracic surgery has received considerable attention [12, 13]. The diaphragm is instrumental in respiration, and preoperative diaphragmatic dysfunction is strongly associated with PPC development [12, 14]. However, to date, no studies have investigated the association between preoperative diaphragmatic function and postoperative survival in patients with lung cancer.

Conventional assessment of diaphragmatic function relies on ultrasonography, which requires specialized equipment and depends on the examiner’s skill and experience, limiting its applicability in routine practice. In contrast, recent studies have shown that the diaphragmatic dome height (DDH), an imaging-based index of diaphragmatic function derived from chest radiography or computed tomography (CT), is associated with dynamic lung hyperinflation, peak VO₂, pulmonary function, health-related quality of life, and exacerbation risk in patients with chronic obstructive pulmonary disease [15, 16].

Chest radiography is a standard component of preoperative evaluation in patients with lung cancer. Therefore, we hypothesized that evaluating diaphragmatic function using DDH on chest radiographs may provide a less invasive and more practical approach for postoperative prognosis prediction. However, no studies have demonstrated an association between DDH and postoperative survival in patients with lung cancer. Accordingly, in this study, we investigated the relationship between preoperative DDH, measured on chest radiographs, and postoperative overall survival (OS) and disease-specific survival (DSS) with respect to respiratory-related deaths in patients with lung cancer and OVD.

## Patients and methods

### Study design and participants

This single-center retrospective cohort study included patients with lung cancer and OVD who underwent video-assisted thoracoscopic surgery (VATS) lobectomy or open thoracotomy at the Department of Thoracic Surgery, Kindai University Hospital, between January 2017 and August 2024. The study outcomes were OS and DSS related to respiratory causes (pneumonia, atelectasis, or respiratory failure). OS was defined as the time from surgery to death from any cause, whereas DSS was defined as the time from surgery to death due to respiratory-related causes, with deaths from other causes treated as competing events.

OVD was defined as a forced expiratory volume in 1 s (FEV₁)/forced vital capacity (FVC) ratio < 70%. VATS was defined as thoracoscopic surgery with an incision length of ≤ 8 cm.

The exclusion criteria were as follows: (1) patients with preoperative phrenic nerve paralysis and (2) patients with preoperative pleural effusion. Specifically, phrenic nerve paralysis was defined as elevation of the hemidiaphragm by more than two intercostal spaces compared with the contralateral side on preoperative chest X-ray. This radiological finding is widely accepted as clinically significant and was verified by board-certified respirologists. All patients received physiotherapy pre- and post-surgery.

This study was approved by the Ethics Committee of the Kindai University Faculty of Medicine (approval number: R06-115) and complied with the ethical standards of the 1964 Declaration of Helsinki and its later amendments. Informed consent was obtained using an opt-out approach, with study details disclosed on the hospital website, under Ethics Committee approval.

### Investigation and evaluation

**Fig. 1:**
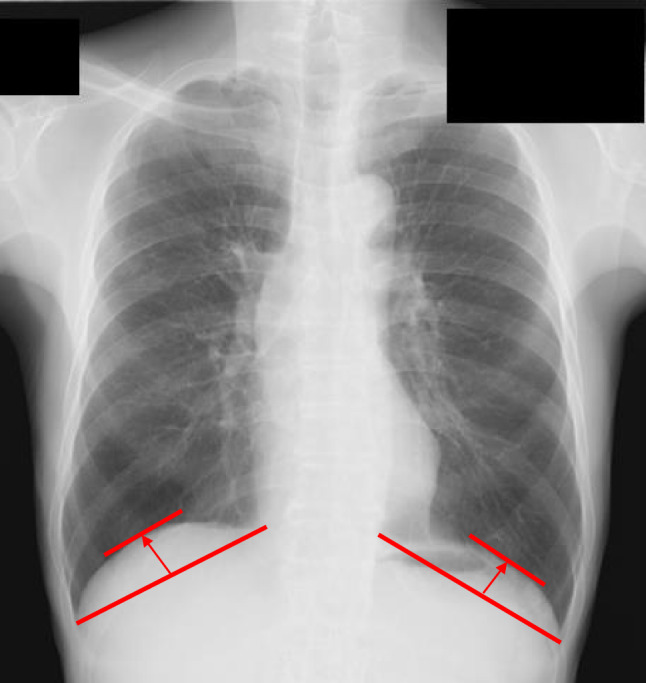
Measurement of diaphragmatic dome height. The height of the right and left diaphragmatic domes was measured using plain chest radiographs acquired within 1 month preoperatively. The measurement was defined as the longest vertical distance from the diaphragmatic silhouette to a line connecting the costophrenic and cardiophrenic angles

The heights of the right and left diaphragmatic domes were measured using chest radiographs captured within 1 month preoperatively. The height was defined as the longest vertical distance from a line connecting the costophrenic angle to the cardiophrenic angle to the diaphragmatic silhouette (Fig. 1) [16]. Measurements were performed using SYNAPSE (Fujifilm Medical, Tokyo, Japan).

PPCs (pneumonia and atelectasis) were evaluated during the postoperative hospital stay using chest radiographs, chest CT imaging, and the Clavien–Dindo classification.

### Statistical analysis

All patients were classified into two groups based on DDH: low DDH (lower than the first quartile [Q1], 18.8 mm) and high DDH (similar or higher than Q1, 18.8 mm), with the DDH on the tumor side used as the representative index. Q1 was selected to objectively classify the lower 25% of the population and avoid potential optimism bias (overfitting) associated with outcome-oriented cutoffs (e.g., receiver operating characteristic analysis) in this single-center cohort. To further validate the appropriateness of this threshold, we performed a sensitivity analysis using restricted cubic spline (RCS) curves.

Between-group comparisons of patient characteristics were performed using the unpaired *t* test, chi-square (χ^2^) test, or Fisher’s exact test, as appropriate. The relationships between DDH and pulmonary function parameters—specifically %VC, FEV_1_/FVC, %FEV_1_, and predicted postoperative (PPO)-%FEV_1_—were evaluated using Pearson’s correlation coefficient. Furthermore, the association between preoperative pulmonary function and postoperative prognosis was assessed using univariate Cox proportional hazards analysis. Two multivariate Cox proportional hazards models (Models 1 and 2) were constructed to control for the effects of potential confounders on the association between DDH and OS. Model 1 was adjusted for age, male sex, BMI, and the following known strong confounders affecting survival outcomes: tumor type, clinical stage (cStage) ≥ 3, CCI, preoperative serum albumin, Brinkman index, %VC, %FEV_1_, surgical approach (open), and DDH < 18.8 mm. Model 2 was adjusted for age, sex, body mass index, Brinkman index, %FEV_1_, cStage ≥ 3, percentage of low-attenuation areas of both lungs (bilateral LAA%), tumor resection site (upper and middle lobes vs. lower lobe), and DDH < 18.8 mm.

To evaluate risk factors for DSS with postoperative respiratory-related death as the outcome, a competing risk analysis was performed using the two Fine–Gray subdistribution hazard models (Models 1 and 2), and both the subdistribution hazard ratios (SHRs) and cumulative incidence were calculated. Respiratory-related death (pneumonia, atelectasis, or respiratory failure) was considered the event of interest, whereas lung cancer recurrence, non-respiratory disease-related death, and accidental death were treated as competing events. Model 1 was adjusted for age, male sex, BMI, and the following known strong confounders affecting survival outcomes: tumor type, cStage ≥ 3, CCI, preoperative serum albumin, Brinkman index, %VC, %FEV_1_, surgical approach (open), and DDH < 18.8 mm. Model 2 was adjusted for age, sex, body mass index, Brinkman index, %FEV_1_, cStage ≥ 3, bilateral LAA%, tumor resection site (upper and middle lobes vs. lower lobe), and DDH < 18.8 mm.

In addition, OS and DSS were estimated using the Kaplan–Meier method, and differences between groups were assessed using the log-rank test. All statistical analyses were performed using JMP software (JMP^®^ Pro, version 18, SAS Institute Inc, Cary, NC, USA) and EZR 2.9.1 (Jichi Medical University, Tochigi, Japan), a graphical user interface for R (The R Foundation for Statistical Computing, Vienna, Austria). It is a modified version of R Commander designed to add statistical functions frequently used in biostatistics. Statistical significance was set at *p* < 0.05.

## Results

**Fig. 2:**
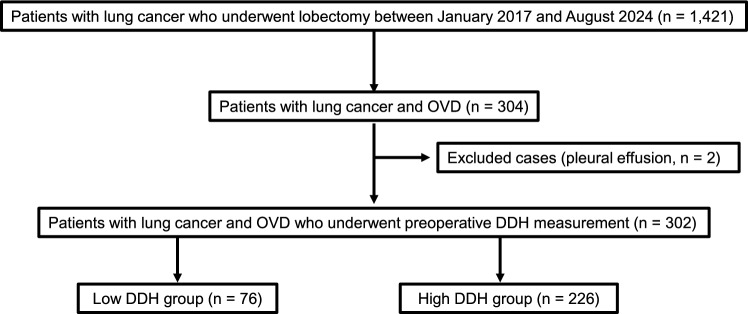
Patient selection flowchart. Between January 2017 and August 2024, 1,421 patients underwent lobectomy. Of those patients, 304 had OVD and underwent preoperative evaluation of DDH. Two patients were excluded due to pleural effusion, which precluded accurate measurement. Overall, 302 patients were included in the final analysis. Among the 302 patients, 76 were in the low DDH group and 226 in the high DDH group. *OVD* obstructive ventilatory disorder, *DDH* diaphragmatic dome height

Between January 2017 and August 2024, 1,421 patients underwent lobectomy, of whom 304 had OVD. Among them, 95 underwent open thoracotomy, and 209 underwent VATS. Two patients with pleural effusion, which made the accurate measurement of DDH impossible, were excluded. Consequently, we included 302 patients in the final analysis: 76 in the low DDH group and 226 in the high DDH group (Fig. 2). Overall, 69 patients (23%) developed PPCs, and 65 patients (21%) died during the postoperative follow-up period. Among the 65 deaths, 14 (21.5%) were cancer-related, 31 (47.7%) were respiratory-related, and 20 (30.8%) were due to other causes. The median follow-up duration was 32 months (IQR, 17–59 months). The highest recorded DDH among the participants was 42.69 mm, whereas the lowest was 7.33 mm (Online Resource 1a, b). Sensitivity analysis using RCS curves revealed a non-linear relationship between preoperative DDH and mortality risk. The hazard ratio increased progressively as DDH decreased below approximately 18.8 mm, which was highly consistent with the predetermined cutoff value of Q1 (18.8 mm) (Online Resource 2).

The patients’ clinical characteristics and comparisons between groups are summarized in Table 1.

**Table 1:** Baseline patient characteristics and comparison between groups

Characteristics	All patients (*N* = 302)	Low DDH group (*n* = 76)	High DDH group (*n* = 226)	*p*-value*
Age (years)	72.9 ± 6.6	73.7 ± 6.8	72.6 ± 6.5	0.19
Male, n (%)	232 (76%)	51 (67%)	181 (80%)	0.02
BMI (kg/m^2^)	22.9 ± 3.3	22.7 ± 4.0	22.9 ± 3.0	0.58
Brinkman index	964.8 ± 757.3	892.0 ± 792.8	989.3 ± 745.3	0.33
Tumor type (Ad/Sq/large/other)	175/32/8/87	41/9/2/24	134/23/6/63	0.87
cStage (I/II/III/IV)	202/51/45/4	45/13/16/2	157/38/29/2	0.15
cT stage (1/2/3/4)	173/71/39/19	40/20/10/6	133/51/29/13	0.76
cN stage (0/1/2/3)	252/32/17/1	61/6/8/1	191/26/9/0	0.04
cM stage (0/1)	298/4	74/2	224/2	0.26
Surgical approach (open/VATS)	95/207	31/45	64/162	0.04
Tumor resection site (upper and middle lobes vs. lower lobe)	201/101	46/30	155/71	0.19
Incision wound (cm)	8.1 ± 4.4	9.0 ± 4.4	7.8 ± 4.3	0.04
Intraoperative bleeding volume (mL)	110.2 ± 166.2	162.0 ± 191.4	92.8 ± 153.5	< 0.01
Operation time (min)	241.4 ± 81.2	258.6 ± 91.3	235.6 ± 76.9	0.03
%VC (%)	100.0 ± 15.2	96.0 ± 14.9	101.3 ± 15.0	< 0.01
FEV_1_/FVC (%)	64.1 ± 7.4	62.1 ± 8.6	64.7 ± 6.8	< 0.01
%FEV_1_ (%)	80.2 ± 16.8	75.9 ± 17.9	81.7 ± 16.3	< 0.01
GOLD classification (I/II/III/IV)	160/128/14/0	32/38/6/0	128/90/8/0	0.05
Bilateral LAA% (%)	16.8 ± 9.7	16.4 ± 9.4	18.0 ± 10.5	0.22
Preoperative serum albumin (g/dL)	4.0 ± 0.4	4.0 ± 0.4	4.1 ± 0.4	0.07
CCI (score)	1.7 ± 1.7	1.8 ± 1.6	1.7 ± 1.7	0.75
PPCs n (%)	69 (23%)	46 (61%)	23 (10%)	< 0.001

Continuous variables are presented as the mean ± standard deviation and were compared using the unpaired *t* test. Categorical variables are presented as n (%) and were compared using the χ^2^ test or Fisher’s exact test

*DDH* diaphragmatic dome height, *BMI* body mass index, *Ad* adenocarcinoma, *Sq* squamous cell carcinoma, *Large* large cell carcinoma, *cStage* clinical stage, *cT stage* clinical tumor stage, *cN stage* clinical nodal stage, *cM stage* clinical metastasis stage, *VATS* video-assisted thoracoscopic surgery, %VC percent vital capacity; *FEV*_*1*_*/FVC* forced expiratory volume in 1 s/forced vital capacity ratio, *FEV*_*1*_ forced expiratory volume in 1 s, *GOLD classification* Global Initiative for Chronic Obstructive Lung Disease classification, *LAA%*, percentage of low-attenuation areas, *CCI* Charlson Comorbidity Index, *PPCs* postoperative pulmonary complications

^*^Comparison between the low and high DDH groups

Compared with the high DDH group, the low DDH group showed significantly higher values for the following variables: male sex (*p* = 0.02), clinical N stage (p = 0.04), surgical approach (open; *p* = 0.04), incision wound (*p* = 0.04), intraoperative bleeding volume (*p* < 0.01), operative time (*p* < 0.01), and PPCs (p < 0.001). In contrast, the low DDH group demonstrated significantly lower values for %VC (*p* < 0.01), FEV_1_/FVC (*p* < 0.01), and %FEV_1_ (*p* < 0.01). No significant differences were observed between the two groups with respect to age, BMI, Brinkman index, tumor type, cStage, clinical T stage, clinical M stage, tumor resection site (upper and middle lobes vs. lower lobe), Global Initiative for Chronic Obstructive Lung Disease (GOLD) classification, bilateral LAA%, preoperative serum albumin, or CCI.

When the relationship between preoperative DDH and pulmonary function parameters was evaluated, statistically significant positive correlations emerged for all parameters examined; however, the strength of these associations was weak, as indicated by the low correlation coefficients. Specifically, DDH showed weak positive correlations with %VC (*r* = 0.12, *p* = 0.03), FEV_1_/FVC (*r* = 0.17, p < 0.01), %FEV_1_ (*r* = 0.16, *p* < 0.01), and PPO-%FEV_1_ (*r* = 0.19, *p* < 0.01). In addition, univariate Cox proportional hazards analyses performed to evaluate the associations between preoperative pulmonary function parameters and postoperative prognosis revealed that none of the preoperative pulmonary function parameters showed a significant association with OS (%VC, *p* = 0.72; FEV_1_/FVC, p = 0.06; %FEV_1_, *p* = 0.87). In contrast, DDH was the only variable that bore a significant association with OS (hazard ratio [HR]: 1.88, *p* < 0.01) (Online Resource 3).

In the Cox proportional hazards model employing survival time as the dependent variable, Model 1 showed that DDH < 18.8 mm (HR: 2.10, 95% confidence interval [CI]: 1.22–3.61, *p* < 0.01), male sex (HR: 3.68, 95% CI: 1.48–9.17, *p* < 0.01), and cStage ≥ 3 (HR: 2.82, 95% CI: 1.49–5.34, *p* < 0.01) were independent prognostic factors for OS. In Model 2, DDH < 18.8 mm (HR: 2.03, 95% CI: 1.18–3.49, *p* < 0.01), male sex (HR: 5.02, 95% CI: 1.98–12.71, *p* < 0.01), cStage ≥ 3 (HR: 3.25, 95% CI: 1.83–5.75, *p* < 0.01), and lower lobe resection (HR: 2.26, 95% CI: 1.35–3.77, *p* < 0.01) remained independent prognostic factors for OS (Table 2).

**Table 2:** Cox proportional hazards model for postoperative overall survival

	Model 1			Model 2		
	HR	95% CI	*p*-value	HR	95% CI	*p*-value
Age	1.00	0.96–1.04	0.91	1.00	0.96–1.04	0.93
Male	3.68	1.48–9.17	< 0.01	5.02	1.98–12.71	< 0.01
BMI	0.98	0.90–1.07	0.71	0.97	0.89–1.05	0.52
Tumor type	1.47	0.65–3.32	0.40	–	–	–
cStage, ≥ 3	2.82	1.49–5.53	< 0.01	3.25	1.83–5.75	< 0.01
CCI	1.23	0.99–1.26	0.05	–	–	–
Preoperative serum albumin	0.67	0.43–1.04	0.08	–	–	–
Brinkman index	1.00	0.99–1.00	0.10	1.00	0.99–1.03	0.08
%VC	0.99	0.97–1.02	0.85	–	–	–
%FEV_1_	1.01	0.99–1.04	0.17	1.01	0.99–1.03	0.18
Surgical approach, open	1.59	0.89–2.81	0.11	–	–	–
Bilateral LAA%	–	–	–	0.99	0.96–1.02	0.66
Tumor resection site (upper/middle [ref] vs lower)	–	–	–	2.26	1.35–3.77	< 0.01
DDH < 18.8 mm	2.10	1.22–3.61	< 0.01	2.03	1.18–3.49	< 0.01

*HR* hazard ratio, *CI* confidence interval, *BMI* body mass index, *cStage* clinical stage, *CCI* Charlson Comorbidity Index, *%VC* percent vital capacity, *FEV*_*1*_ forced expiratory volume in 1 s, *LAA%* percentage of low-attenuation areas, *ref* reference, *DDH* diaphragmatic dome height

**Fig. 3:**
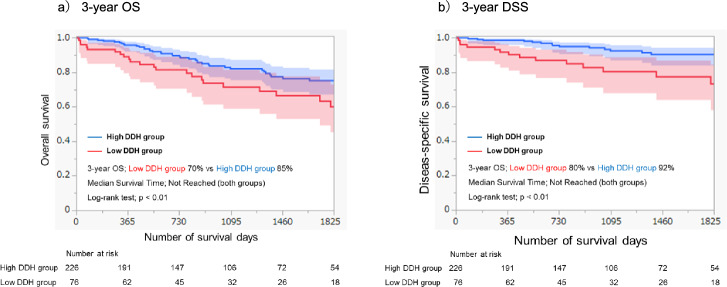
Kaplan–Meier curves for 3-year OS (**a**) and 3-year DSS (**b**) postoperatively. **a** Patients in the low DDH group had significantly worse 3-year OS compared with those in the high DDH group (70% vs 85%, p < 0.01). **b** 3-year DSS, with postoperative respiratory-related death as the outcome, was lower in the low DDH group than in the high DDH group (80% vs 92%, p < 0.01). *DDH* diaphragmatic dome height, *OS* overall survival, *DSS* disease-specific survival

Furthermore, Kaplan–Meier analysis and the log-rank test showed that patients in the low DDH group had significantly lower 3-year OS (low DDH group: 70% vs high DDH group: 85%,* p* < 0.01; Fig. 3a) and 3-year DSS (low DDH group: 80% vs high DDH group: 92%, *p* < 0.01; Fig. 3b) than those in the high DDH group.

**Fig. 4:**
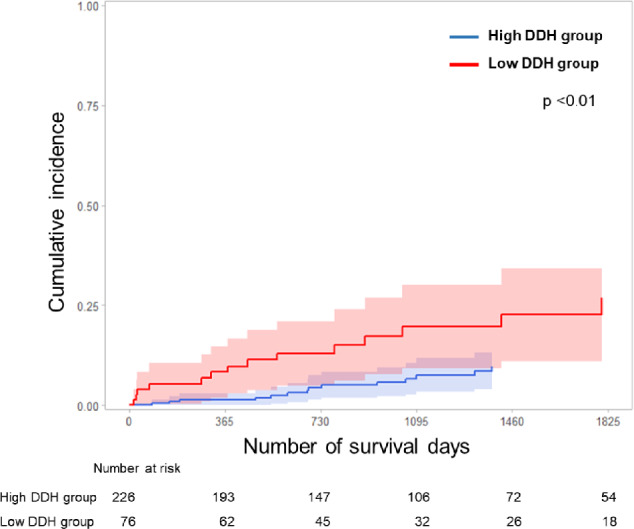
3-year cumulative incidence of postoperative respiratory-related death. The 3-year cumulative incidence of postoperative respiratory-related death was 20% in patients in the low DDH group and 5% in those in the high DDH group (p < 0.01). *DDH* diaphragmatic dome height

In addition, a competing risk analysis using the Fine–Gray proportional hazards model was performed to evaluate the 3-year cumulative incidence and risk factors for DSS. The 3-year cumulative incidence of postoperative respiratory-related death was 20% in the low DDH group and 5% in the high DDH group (*p* < 0.01; Fig. 4). In Model 1, DDH < 18.8 mm (SHR: 2.50, 95% CI: 1.14–5.45, *p* < 0.05) and cStage ≥ 3 (SHR: 3.33, 95% CI: 1.40–7.92, *p* < 0.01) were identified as independent risk factors for postoperative respiratory-related death. In Model 2, DDH < 18.8 mm (SHR: 2.31, 95% CI: 1.20–4.42, *p* < 0.01), male sex (SHR: 3.77, 95% CI: 1.14–12.5, *p* < 0.05), cStage ≥ 3 (SHR: 4.25, 95% CI: 1.96–9.20, *p* < 0.01), and lower lobe resection (SHR: 2.51, 95% CI: 1.18–5.34, *p* < 0.01) remained independently associated with postoperative respiratory-related death (Table 3).

**Table 3:** Fine–Gray competing risk analysis for postoperative respiratory-related mortality

	Model 1			Model 2		
	SHR	95% CI	*p*-value	SHR	95% CI	*p*-value
Age	1.03	0.96–1.10	0.37	1.02	0.96–1.08	0.38
Male	2.86	0.90–9.07	0.07	3.77	1.14–12.50	0.03
BMI	0.96	0.87–1.07	0.54	0.99	0.89–1.10	0.89
Tumor type	0.95	0.70–1.27	0.73	–	–	–
cStage, ≥ 3	3.33	1.40–7.92	< 0.01	4.25	1.96–9.20	< 0.01
CCI	1.14	0.95–1.36	0.14	–	–	–
Preoperative serum albumin	0.68	0.38–1.19	0.18	–	–	–
Brinkman index	1.00	0.99–1.00	0.94	1.00	0.99–1.00	0.78
%VC	1.02	0.98–1.06	0.30	–	–	–
%FEV_1_	0.98	0.95–1.01	0.29	0.98	0.96–1.01	0.28
Surgical approach, open	0.60	0.21–1.68	0.34	–	–	–
Bilateral LAA%	–	–	–	1.02	0.98–1.06	0.23
Tumor resection site (upper/middle [ref] vs lower)	–	–	–	2.51	1.18–5.34	< 0.01
DDH, < 18.8 mm	2.50	1.14–5.45	0.02	2.31	1.20–4.42	< 0.01

*SHR* subdistribution hazard ratio, *CI* confidence interval, *BMI* body mass index, *cStage* clinical stage, *CCI* Charlson Comorbidity Index, *%VC* percent vital capacity, *FEV*_*1*_ forced expiratory volume in 1 s, *LAA%* percentage of low-attenuation areas, *ref*, reference, *DDH* diaphragmatic dome height

## Discussion

The present study showed that, among patients with lung cancer and OVD, lower preoperative DDH was significantly associated with reduced OS and DSS due to respiratory-related deaths. These findings suggest that DDH, as assessed on routine chest radiographs, may serve as a useful prognostic indicator for long-term survival following lung cancer surgery, independent of previously reported predictors such as pulmonary function, tumor stage, and surgical invasiveness. To the best of our knowledge, this is the first study to demonstrate an association between preoperative DDH and OS and DSS in patients with lung cancer and OVD, based on a widely available and standard imaging modality in daily clinical practice.

To date, no study has directly investigated the impact of preoperative diaphragmatic function on postoperative survival in lung cancer. However, its influence can be explained through several mechanisms, including impaired exercise tolerance pre- and postoperatively, increased risk of PPCs, and challenges in the continuation of adjuvant therapies. First, hyperinflation, a characteristic feature of patients with OVD, leads to flattening of the diaphragm, which correlates with reduced pulmonary function, increased dyspnea, and impaired diaphragmatic contractility [11]. In general, diaphragmatic dysfunction is closely associated with decreased exercise tolerance and physical activity [11]. In patients with lung cancer and diaphragmatic dysfunction, diminished ventilatory efficiency and increased respiratory workload may accelerate physical frailty and sarcopenia during the postoperative course [17]. Consequently, loss of skeletal muscle mass and strength further impairs exercise tolerance, leading to deterioration in the overall condition. Reduced exercise tolerance is strongly correlated with diminished performance status (PS) [18], which has been identified as a primary reason for discontinuation or ineligibility for adjuvant chemotherapy or immunotherapy [19]. This may result in higher recurrence rates and reduced survival. Thus, diaphragmatic dysfunction may adversely affect PPC occurrence, systemic condition, and treatment continuity, making it a clinically significant determinant of long-term prognosis.

Second, as the primary inspiratory muscle, the diaphragm is essential in ventilation. Diaphragmatic dysfunction is associated with reduced ventilatory efficiency and impaired cough strength, which contributes to impaired sputum clearance and hypoventilation, thereby increasing the risk of PPCs [14]. Development of PPCs is associated with prolonged hospitalization, increased medical costs, delayed recovery, higher readmission rates, and poorer postoperative survival [6, 20]. In this study, lower DDH was identified as an independent risk factor for respiratory-related death, further supporting the prognostic importance of diaphragmatic dysfunction.

Our key finding is that DDH, derived from routine chest radiography—a standard, widely available, and cost-effective imaging modality—was independently associated with long-term prognosis in patients with lung cancer, beyond conventional predictors such as tumor stage, pulmonary function, and surgical approach. Thus, DDH may serve as an indicator of PPC risk as well as novel and practical prognostic marker for long-term survival following lung cancer surgery. Traditionally, diaphragmatic function has been evaluated using ultrasonography to measure diaphragmatic thickness and mobility [21], which correlates with DDH [16]. However, ultrasound assessment is limited by operator dependency, reproducibility, and equipment availability, restricting its applicability across all clinical settings. In contrast, chest radiography is routinely performed as part of preoperative evaluation cod all patients with lung cancer, and DDH can be assessed without additional cost, radiation exposure, or equipment. Our findings highlight that DDH derived from chest radiographs is a clinically meaningful and universally applicable prognostic indicator, feasible across diverse clinical environments. Moreover, combining DDH with existing risk factors such as pulmonary function, nutritional status, tumor stage, and exercise tolerance may allow for more refined preoperative risk stratification, improved prediction of PPCs, and tailored perioperative management strategies.

We also investigated the relationship between DDH and preoperative pulmonary function. Although statistically significant correlations were observed, the correlation coefficients were weak (r < 0.2), suggesting that the clinical relevance of these associations is limited. Furthermore, in the univariate analyses, standard pulmonary function parameters (e.g., %VC and %FEV_1_) were not significantly associated with postoperative survival, whereas low DDH was a strong predictor of poor prognosis (HR: 1.88, *p* < 0.01). Although earlier studies have reported associations between impaired pulmonary function and postoperative mortality [22], recent advances in perioperative management and stricter patient selection may have attenuated the prognostic impact of conventional spirometric parameters, placing greater emphasis on postoperative pulmonary complications [6, 20], physical reserve, and functional capacity [9, 18]. In this context, DDH may serve as a more sensitive surrogate marker of the physiological reserve. It may reflect the functional aspects of the diaphragm and respiratory muscle weakness that cannot be fully captured by spirometry alone.

Another notable finding of this study was the seemingly paradoxical relationship between smoking history and DDH. Smoking has been reported to induce emphysematous changes in the lung, leading to diaphragmatic flattening [11]. However, the lower Brinkman index observed in the low DDH group in the present study suggests that phenotypic differences may have influenced this result. Smoking-related lung disease is clinically considered to comprise at least two major phenotypes: one predominantly characterized by emphysematous lesions and the other by peripheral airway–dominant disease [23]. In the former phenotype, lung hyperinflation is a principal feature and likely to result in diaphragmatic flattening. In contrast, in the latter phenotype, airway obstruction is the predominant pathological feature, and lung hyperinflation is not necessarily prominent even in patients with a heavy smoking history; consequently, DDH tends to be preserved [24]. The higher Brinkman index observed in the high DDH group may therefore be attributable to the greater proportion of patients with airway-dominant disease without significant hyperinflation. Conversely, the lower Brinkman index in the low DDH group suggests that reduced DDH in these patients may not be primarily driven by smoking-related lung hyperinflation, but rather by non-smoking-related factors affecting diaphragmatic function, such as respiratory muscle weakness or sarcopenia.

This study had some limitations. First, this was a single-center study, and caution is warranted in generalizing the findings to broader clinical practice. Second, preoperative respiratory muscle strength and exercise capacity, which may also affect survival, were not evaluated using indices such as maximal inspiratory/expiratory pressures or peak VO_2_ and 6-min walk distance. However, as these measures are strongly associated with diaphragmatic function and correlate with DDH, the impact of this limitation on the results is likely minimal. Third, although preoperative sarcopenia and frailty may influence diaphragmatic function and survival, specific indicators of sarcopenia and frailty were not incorporated. Nonetheless, the study population maintained PS 0–1, and no cases of severe malnutrition based on preoperative albumin levels were observed, suggesting a limited influence on outcomes. Fourth, postoperative changes in PS and diaphragmatic function over the follow-up period were not assessed. In reality, postoperative complications, recurrence, and age-related functional decline may all affect PS and diaphragmatic function, with significant implications for long-term survival and quality of life. Finally, our study cohort included patients with advanced-stage lung cancer. Although invasive procedures associated with advanced disease inevitably increase the risk of postoperative mortality, we minimized this bias by adjusting for cStage and surgical approach in our multivariate analyses. Nevertheless, the potential influence of tumor progression and surgical invasiveness cannot be completely ruled out. Future studies restricted to patients with early-stage lung cancer are warranted to rigorously validate the direct association between DDH and respiratory-related death. Future studies evaluating longitudinal changes in PS and diaphragmatic function are needed to provide a more comprehensive understanding of long-term outcomes.

In conclusion, preoperative DDH was identified as an independent risk factor for OS and DSS due to respiratory-related deaths in patients with lung cancer and OVD undergoing lobectomy. Patients with lower DDH had significantly poorer survival than those with higher DDH, suggesting that reduced DDH reflects hyperinflation, diminished respiratory reserve, and impaired exercise tolerance, and may serve as a novel physiological marker of long-term prognosis. DDH assessment using preoperative chest radiography is non-invasive, readily available, and universally applicable, complementing established risk factors such as age, pulmonary function, and comorbidities. Therefore, DDH aids in individualized perioperative management and postoperative follow-up strategies. Specifically, patients with low DDH may benefit from intensified perioperative rehabilitation, enhanced respiratory management, tailored surgical approaches, and closer postoperative monitoring.

Future multicenter and prospective studies are required to validate the generalizability and predictive accuracy of DDH. Incorporating DDH into comprehensive risk stratification models, together with other clinical indicators, may improve the quality of clinical decision-making and contribute to better long-term outcomes in patients with lung cancer and OVD.

### Glossary

%FEV_1,_ percent FEV_1_; %VC, percent vital capacity; 95% CI, 95% confidence intervals; BMI, body mass index; CCI, Charlson Comorbidity Index; cStage, clinical stage; CT, computed tomography; DDH, diaphragmatic dome height; DSS, disease-specific survival; FEV_1_/FVC, forced expiratory volume in 1 s/forced vital capacity; HR, hazard ratios; OS, overall survival; OVD, obstructive ventilatory disorder; PPC, postoperative pulmonary complications; PS, performance status; SHR, subdistribution hazard ratios; VATS, video-assisted thoracoscopic surgery.

## Data Availability

No generative AI or AI-assisted technologies were used in the writing or editing of this manuscript. The datasets generated during the current study are available from the corresponding author on reasonable request.
